# Probing key informants’ views of health equity within the World Health Organization’s Urban HEART initiative

**DOI:** 10.1186/s12889-022-14395-z

**Published:** 2022-10-31

**Authors:** Michelle Amri, Patricia O’Campo, Theresa Enright, Arjumand Siddiqi, Erica Di Ruggiero, Jesse Boardman Bump

**Affiliations:** 1 Takemi Program in International Health, Harvard T.H. Chan School of Public Health, 665 Huntington Avenue, Bldg. 1, 02115-6021 Boston, MA United States of America; 2 Dalla Lana School of Public Health, University of Toronto, 155 College Street, M5T 1P8 Toronto, ON Canada; 3 Li Ka Shing Knowledge Institute, St. Michael’s Hospital, 209 Victoria Street, M5B 1T8 Toronto, ON Canada; 4 Department of Political Science, University of Toronto, 100 St George Street, M5S 3G3 Toronto, ON Canada; 5 Gillings School of Global Public Health, University of North Carolina - Chapel Hill, Chapel Hill, USA; 6 Bergen Centre for Ethics and Priority Setting, University of Bergen, Bergen, Norway

## Abstract

To date, no studies have assessed how those involved in the World Health Organization’s (WHO) work understand the concept of health equity. To fill the gap, this research poses the question, “how do Urban Health Equity Assessment and Response Tool (Urban HEART) key informants understand the concept of health equity?”, with Urban HEART being selected given the focus on health equity. To answer this question, this study undertakes synchronous electronic interviews with key informants to assess how they understand health equity within the context of Urban HEART. Key findings demonstrate that: (i) equity is seen as a core value and inequities were understood to be avoidable, systematic, unnecessary, and unfair; (ii) there was a questionable acceptance of need to act, given that political sensitivity arose around acknowledging inequities as “unnecessary”; (iii) despite this broader understanding of the key aspects of health inequity, the concept of health equity was seen as vague; (iv) the recognized vagueness inherent in the concept of health equity may be due to various factors including country differences; (v) how the terms “health inequity” and “health inequality” were used varied drastically; and (vi) when speaking about equity, a wide range of aspects emerged. Moving forward, it would be important to establish a shared understanding across key terms and seek clarification, prior to any global health initiatives, whether explicitly focused on health equity or not.

**Supplementary Information:**

The online version contains supplementary material available at 10.1186/s12889-022-14395-z.

## Introduction

Despite heightened attention paid to health inequity—which is often understood as unfair and unjust differences [17, as opposed to inequalities which are systematic measured differences—how the concept is defined and operationalized varies [1]. This is further complicated with the interchanged use of terms, such as between “health inequity”, “health inequalities”, and “health disparities” [2, 3], or “inequity” and “inequality” [4].

This can be particularly problematic for major players, such as the World Health Organization (WHO), that set norms and standards in the field. A scoping review [5], following systematic methods [6], determined that there are no empirical analyses conducted that investigate the World Health Organization’s (WHO) approach to health equity. Following this study, recent analysis investigated key texts produced by the WHO in the domains of health promotion, the social determinants of health (SDH), and urban health, to understand how “equity” has been conceptualized [7]. Among other findings, this study found that “despite expressing a distinction between ‘inequities’ and ‘inequalities,’ there are several instances where the WHO uses the terminology of ‘inequity’ and ‘inequality’ interchangeably” [7]. This work is beneficial for understanding discourses within the WHO texts and how these WHO texts approached the concept of equity. This is valuable because these discourses are anticipated to be subsequently acted upon by the WHO and by other global and public health bodies and operationalized into policy and practice in member states, given the authoritative position of the WHO and influence of its work.

However, to the best of our knowledge, there are no such studies investigating how actors involved in health equity work of the WHO understand health equity, which is what our study does. Therefore, we assessed how equity is conceived by those involved the Urban Health Equity Assessment and Response Tool (Urban HEART), a WHO initiative focused on health equity, as it can shed light on where misunderstandings and discrepancies lie among those who are immersed in the field.

Urban HEART was born out of the Commission on Social Determinants of Health [8, 9] and was designed as a tool for cities to address health inequities [10]. While Urban HEART was developed from 2007 to 2010 [11, 12] and is no longer largely institutionally supported through the WHO Kobe Centre (WKC), also known as the WHO Centre for Health Development, it has been used in over 100 cities, including Barcelona, Detroit, Matsapha, Tehran [11], and Toronto [13]. Urban HEART helps cities *assess* priority areas and *respond* through action to combat inequity through focusing action on social, economic, and environmental determinants of health [11, 14]. *Urban HEART: Urban Health Equity Assessment and Response Tool* [14], the main Urban HEART text, not only provides an overview of concepts and principles—including on inequity in health—but it introduces Urban HEART and provides the rationale and expected achievements through using Urban HEART. This text lays out an understanding that “equity in health implies that ideally everyone could attain their full health potential and that no one should be disadvantaged from achieving this potential because of their social position or other socially determined circumstance” [14]. However, this differs from the views of others involved in Urban HEART, such as those who see inequities as being systematic inequalities that are socially produced, entailing they are unjust and modifiable [8]. Further, its approach to equity in health is broad, with Urban HEART including indicators on: health care outcomes, health determinants, physical environment and infrastructure, social and human development, and economics [14].

Urban HEART was selected for analysis due to its focus on health equity and this analysis is rooted in an understanding of the prominence of the WHO. In other words, that these discourses within the WHO diffuse into the work of city and national officials, as well as academics and researchers, all of which were also responsible for developing Urban HEART.

## Methods

### Study design

We employed a qualitative research design to answer the research question, “how do Urban HEART key informants understand the concept of health equity?” Synchronous electronic interviews were conducted by MA with key informants, employing open-ended questions to enhance the likelihood that the informants would not conform their responses to only what was being investigated.

Synchronous electronic interviews were selected given the vast geography of informants, as it is infeasible to travel to numerous cities to conduct interviews, particularly during the COVID-19 pandemic. Additionally, synchronous interviews were selected over asynchronous interviews (which do not occur in real time), as they allow for assessing non-verbal cues, reduce the perception of researcher laziness leading to brevity, and can allow for additional clarity and precision in prompt follow-ups through probing [15].

Structured interviews (i.e., following a set of predetermined questions available in the supplemental file 1) were preferred to semi-structured interviews to ensure all participants were answering the same questions, and therefore facilitating enhanced data analysis through comparing views. However, probing was undertaken to ask additional follow-up and clarification questions. This study was approved by the Health Sciences Research Ethics Board at the University of Toronto (protocol number: 39543).

### Data collection

Data from 18 key informants who were involved in Urban HEART were collected, including those employed by the WHO as technical officers, civil servants, and researchers. Selection criteria for key informants included those with experience with Urban HEART, through working on Urban HEART, including those who guided the technical direction of Urban HEART, drafted key reports, or acted as focal points for member states at the WKC; implementing Urban HEART in a city; using Urban HEART as a tool of analysis after implementation in a city; using WKC Urban HEART funding to apply the methodology and secure policy change; and any combination of the above. Thus, the aim was to get a multi-faceted perspective of how health equity is understood. The sampling strategy was designed to glean a global perspective, thus placing no geographical restrictions on where informants had worked on Urban HEART. Informants had worked on implementing Urban HEART within the Americas, Eastern Mediterranean, and European regions (regions as defined by the WHO). There were also informants from the WKC who were involved in supporting implementation or acting as a focal point across all the regions of the WHO. The breakdown of informants who are technical officers employed by the WHO, civil servants, researchers, others, and those who had overlapping identities, in addition to the locations of key informants, are intentionally not specified to ensure individual identities are not easily decipherable. However, to provide a generalized understanding of key informants, half of the study participants were not employed by the WHO on a full-time basis, whereas the remaining half were.

Interviews were conducted from August 2020 to January 2021 (n = 16) and two additional informants opted to type their responses to questions due to language barriers. Responses from four informants were excluded from analysis due to their limited involvement with Urban HEART. Two of these informants were researchers applying Urban HEART as a method of analysis with the aim of presenting their findings to government to institutionalize Urban HEART, but the tool has not yet been employed within their respective cities. The third informant is a researcher who attended an Urban HEART workshop; however, they indicated there is no evidence of Urban HEART in their respective country following this workshop. And the fourth informant who was excluded from analysis is a civil servant in a ministry of health working on environmental and occupational health, and despite answering questions about Urban HEART, did not indicate if and how it was used. These latter two informants had both typed and submitted responses to questions due to language barriers, so further questioning was difficult. As such, 14 informants’ interviews were included in this analysis.

These informants were contacted through connecting with authors of journal articles on Urban HEART, purposeful sampling of contacts to the research team, and snowball sampling. Potential participants were sent an interview consent form through email, which detailed the goal of the study, provided contact information for the researchers, and stated participation was voluntary. Once a participant signed the consent form, a structured, synchronous electronic interview (i.e., live interview online through Zoom) was scheduled and undertaken.

### Data analysis

Once an interview was conducted, it was transcribed to facilitate data analysis. NVivo12 was used to store transcripts and undertake analysis. Transcripts were thematically analyzed using both a priori and inductive codes, with the former focused on assessing usage of terms (e.g., health inequity versus inequality). Interviews and analysis ceased after saturation was achieved through the perspective of *data* saturation described by Sandelowski, where there is “informational redundancy,” meaning no new information will be presented [16].

## Results

In seeking to determine how Urban HEART key informants understand the concept of health equity, it became evident this was multi-faceted. These findings signal an unclear concept of health equity, the use of associated terms, what it refers to, among others. These findings are presented briefly in Table 1 and discussed at length in the section below.

**Table 1:** Summary of key results

Finding	Summary
Equity as a core value and understanding inequity as avoidable, systematic, unnecessary, and unfair	Informants felt equity was a core value and understood inequity as avoidable, systematic, unnecessary, and unfair.
Questionable acceptance of need to act	However, despite understanding the above aspects of health inequity and using this vocabulary, the language around “unnecessary” was found to be politically sensitive and inhibited uptake of policy aimed at improving health equity, which is further discussed below under “questionable acceptance of need to act.”
Health equity as vague	Health equity as a concept was expressed as being vague, both in its conception and operationalization. Respondents elaborated, describing that while “health equity” was understood as a philosophical term rooted in morals or ethics, “health inequity” and “health inequality” seemed to be understood more easily due to their quantifiable nature. This could be due to the nature of Urban HEART, which requires quantitative expertise, potentially “biasing” respondent understandings in this way.
Country differences	According to key informants, this recognized vagueness inherent in the concept of health equity may be due to its rooting in social justice and in seeking to ensure health and well-being, which by nature is difficult to define, and/or that countries may understand the concept of health equity differently. This latter point about countries understanding health equity differently may also be linked to utilizing terms differently when referring to the same thing.
Health inequality, health inequity, and health equity: differences across terms	While this above point about country differences was shared by informants, they themselves used the terms “health inequity” and “health inequality” differently. These differing understandings placed: (i) inequalities as measurable and inequities as philosophical; (ii) inequalities as differences but inequities as inequalities that can be addressed; and (iii) addressing inequalities as entailing equal provision, whereas addressing inequities necessitates being unequal but fairer. Further to this, (iv) a differentiation was made between “equity” and “health equity” by two informants. However, there is a possibility that policymakers may intentionally inhibit political action by pointing to both the vagueness of health equity and debating terms like “unnecessary” to perpetuate the status quo.
Health inequities in what?	Overall, it appears there is no uniform or shared understanding of these terms across informants, and equity was referred to in terms of a similarly wide range of aspects.

### Equity as a core value and understanding inequity as avoidable, systematic, unnecessary, and unfair

The notion of equity as a core value and being key for health was readily apparent, particularly when informants were asked the question, “in your own work, how do you think about the relationship of equity and health?” This is simplistically stated by Informant 13, who indicated, “I believe that equity is a core value.” This informant also elaborated to specify the relationship of this value to the work of the WHO, by stating that the:

*“WHO definitely makes that very clear in its work that all people have the right to health. And so, in that statement is implied that you know, equitable access to opportunities for health for health, for healthcare. The full range of healthcare that has to be insured so yeah equity is a core of WHO’s work and a core value in the work that I do on a daily basis”* (Informant 13).

In addition to equity being considered “core,” informants also understood that inequity is avoidable, systematic, unnecessary, and unfair, which aligns with the widely used Whitehead definition [17]. For example, one informant indicated that “so you want to have a fair and, of course, all those avoidable health inequalities in a community, in a city or in a country are being addressed or by the government” (Informant 3).

And when an informant was asked about relevant theories on justice or inequality, during which the informant asked for clarification, the interviewer reapproached the question by stating in terms of what to address, whether this was resources or other focuses. The informant then answered the question by discussing Urban HEART where this similar understanding of inherent values of fairness shines through.

*“It was derived on fairness and it was much beyond just looking at differences in groups. It was being able to say that they are systematic, that they are unfair. And we had to characterize why it is unfair, and that something could be done about them and it had. So largely over-simplified it but that was also kind of how we introduce the Urban HEART concept to people as to why we want to fix this, because it’s fixable”* (Informant 6).

And similarly reflected in the rationale for how Urban HEART was operationalized, as “[…] they did sampling according to neighbourhoods, because it was fair, and neat to know about the neighbourhoods’ health conditions” (Informant 5).

### Questionable acceptance of need to act

Political sensitivity arose with respect to acknowledging inequities using the Whitehead [17] criteria of “unnecessary.” This is nicely expressed through an informant’s discussion of the reception of Urban HEART by government:

*“when we were submitting our staff reports to Council about the goal of the [retracted], which was our name for Urban HEART and what kind of inequities we wanted to tackle. The word was always ‘tackle,’ right, and there were these three dimensions that it was that they were systematically produced, they’re modifiable, they’re fair. So, there’s a lot of discussion about whether or not we should include; oh, I know, it was systematic, unnecessary, and unfair. And there was a fair amount of discussion around whether or not we should be including the concept of unnecessary. Why am I saying this? That was sort of the sensitive area for the relationship between theory and political decision making you’ve kind of touched because, well, I don’t really know why. But there was always a lot of pushback that should we really be saying that these are unnecessary. I think it was too sensitive. People could accept unfair, unsystematic. But there would always be these debates, well it’s unnecessary, but it is necessary because you know we’re doing. I don’t know. I can’t really say”* (Informant 10).

When probed on how the terminology used was determined, the informant indicated it was their “plain language understanding of Whitehead’s definition of what health inequities was” (Informant 10).

In this above quote, it is clear that Whitehead’s [17] original definition of health inequity has made it easier for governments and other relevant stakeholders to understand key aspects of inequity (“systematic, unnecessary, and unfair”) through characterizing health inequities in this way to be able to contrast this with health inequalities. However, the nature of these criteria is difficult to define and set bounds on, which perhaps makes it difficult for policymakers to acknowledge and act on. While this may be unintentional, it can also be intentional, as a way for governments to shirk their responsibilities and “requirement” to act accordingly. This may be due to the difficulty of addressing “causes of causes” or the root causes of ill health, the need to address the privileges or power of certain groups, the preference to prioritize other public policy issues over health and well-being, or other reasons. This is also reflected in another informant’s discussion of the hesitancy for countries to recognize inequity,

*“countries really didn’t want to discuss equity and a lot of the countries, let’s say China and others would say we’re very equitable, you know, so it would be not kind of denial, but it was a political thing, you could not say you were not equitable, politically that was very dangerous for the respective countries”* (Informant 7).

Conversely, these illustrations of government hesitancy to recognize and address inequity were not expressed by all. For example, one informant expressed that, “I think because everybody per se is against inequality, everybody. Nobody can say inequality is alright actually, to me. Nobody can accept, even policymakers. Because it’s unacceptable to everybody” (Informant 1).

It is noteworthy that Informant 10, who indicated that the concept of inequity may have been sensitive in the political arena, expressed the value of Urban HEART: “well, I just don’t think anyone disagreed with it, with the concept. I think it was a really incredibly valuable tool for educating decision-makers about the link between health and determinants of health” (Informant 10). And similarly, Informant 1, who indicated their belief in the widespread acceptance that everyone is against inequality, expressed similar sentiments about Urban HEART: “but you know, the concept is very much admirable. It’s very wise, it’s logic, you’re measuring the inequalities […]” (Informant 1).

### Health equity as vague

Despite this broader understanding of the key aspects of health inequity as being avoidable, systematic, unnecessary, and unfair, the concept of health equity was seen as vague. One informant acknowledged that “health equity is honestly a very vague concept” (Informant 1), and for this reason stated they preferred to use the terms “health inequity” or “health inequality.” Another informant similarly alluded to the vagueness of health equity: “so it’s understanding health inequity, because when you come to think of it, it’s like, ‘okay, what’s that?’ Health inequity is so deep or the concept is something that needs to be fine-tuned” (Informant 3). When probed on this notion, the informant emphasized the importance of having a definition early on which, in their understanding was:

*“unfair and avoidable differentials or regions that people have either when you were born, when you’re growing up, living or surviving, when you’re already learning and when you’re working or when you grow up as an adult, that these differentials actually should not be there because people need to have equivalent access to health services and that things should be fairly distributed, whether it will be income, whether it’s like recognition, for example […]”* (Informant 3).

Contrasting understandings of “health equity,” “health inequity,” and “health inequality,” it appears the quantifiable nature of the latter two means they are more tangible. This is in opposition to “health equity,” which is understood as a matter of morals or ethics. However, this perception could be due to the nature of Urban HEART requiring quantitative expertise, potentially “biasing” respondent understandings in this way. Further, “health inequity” and “health inequality” are understood as “negative” definitions, whereby an improvement should or can be made. Conversely, the “positive” nature of “health equity” renders the output or outcomes sought intangible. Thus, this positive nature of health equity makes policy goals and objectives difficult to define.

### Country differences

According to key informants, the recognized vagueness inherent in the concept of health equity may be due to various factors. One may be because of its rooting in social justice and in seeking to ensure health and well-being, which by nature is difficult to define. Informant 7 summarized this through expressing:

I mean, the notion of social justice, it’s going to vary from country to country, in some ways, as well. I mean, I think that, you know, some of the core human equity issues are based on the human rights chart. Some of them are based actually on the WHO concepts in a sense of assuring not only that, you know? What is the absence of health. It’s also ensuring social well-being, well, what does that mean? And they’re still struggling to define that in some ways.

Also reflected in this above quote by Informant 7 is a country-specific understanding of social justice and, subsequently, health equity. This relationship between different countries’ understanding of social justice and health equity was further emphasized by the same informant by saying, “I mean, the notion of social justice, it’s going to vary from country to country, in some ways, as well” and that:

*You know there’s this notion of equity. However, that’s you know what values mean in Japan and Asia and China is very different than what it means in the United States, which is very different, what it means in Canada.*”^1^

Evidently, informants believe there are geographical differences, but further information is needed to assess differences in how equity might be conceived in different places based on context-specific political rationalities, cultures, institutional frameworks, etc.

This was also reflected by other informants; for example, Informant 6 indicated:

*the thinking that comes from the way Canada thinks and some of the Nordic countries think or Australia, New Zealand, you know, there’s a lot of health equity discussion in these countries, and that is not the same in any other country. They don’t see it either intellectually the same or in practice they don’t see it the same. It took a long time for the U.S. to start talking about social determinants of health and it was a shock when they did. Because although they [U.S.] were part of the Commission on Social Determinants of Health, they never really talked about health equity and social determinants of health, but they also picked up the language, five to seven years ago*.

This informant’s example of the U.S. begins to shed light on the role of the political determinants of agenda setting; in particular, how ideologies influence the uptake of health equity as a concept. Evidently, countries can be involved in a global process, but it takes time for the ideas to be taken up within their respective borders.

This difference in understanding and/or weight given to health equity has implications for the WHO. Informant 7 touched on this by indicating:

from a WHO standpoint, it’s not that there are basic values, but they are very, very different. And I think it’s always a very big important issue for WHO to understand where they put a lot of, not to overuse the word value, but a lot of credence on the fact that respect for the fact that there are going to be these very important contextual and cultural differences in different countries.

But it is not only that countries may understand the concept of health equity differently, but also that countries may utilize terms differently when referring to the same thing, which is explored in depth in the section below. This is expressed by an informant when discussing the difference between “inequality” and “inequity,” where Informant 4 stated, “there’s like a terminology issue that depending on if it’s from U.K., or, but yeah.”

Further, there is a possibility that policymakers may use the vagueness of health equity and political sensitivity of terms such as “unnecessary” to intentionally inhibit political action. However, this study did not provide evidence to support this position.

### Health inequality, health inequity, and health equity: differences across terms

How the terms “health inequity” and “health inequality” were used varied drastically. These differing understandings placed: (i) inequalities as measurable and inequities as philosophical; (ii) inequalities as differences but inequities as inequalities that can be addressed; and (iii) addressing inequalities as entailing equal provision whereas addressing inequities necessitates being unequal but fairer. Further to this, (iv) a differentiation was made between “equity” and “health equity” by two informants. While these differing understandings are touched on below, it is of note that there is no uniform or shared understanding of these terms. This is surprising given that informants in the study referred to the key characteristics of health inequities outlined by Whitehead [17] and, in general, how widely taken up the Whitehead definition is. It is notable that despite these informants’ experiences with Urban HEART, where the focus is health equity, there was so much variability in the understanding of the term. This variance across understanding “health equity” is problematic for many reasons, most obvious being that it can lead to different understandings of where or how to act:

(i) One informant understood the difference being that an inequality is “measurable,” whereas inequity is a “*philosophic* term” (emphasis added) (Informant 1). This informant has indicated that:

*“health inequality is much more measurable in our vision. Because philosophically, it’s possible to allocate some budgets less than the others to some groups. But you can consider that this is equitable. But for this purpose, I urged everybody to work on health inequalities, not health equity or health inequity”* (Informant 1).

This seemed to align with other informants’ views, which is illustrated in discussing what Urban HEART sought to investigate. Informant 5 indicated that “we could see how *inequality* in [retracted] health was visible in the data,” but also that “the goal of that was to show *inequity* in a city that is representative of the country” (emphasis added for both quotes). And similarly, Informant 13 indicated “well, I think the ultimate goal is to reduce health inequities, promote health equity in the community.” However, this understanding did not align with Informant 10, who stated “[…] I mean the tool was really focused on measuring inequities.”

(ii) Another informant specified that inequalities that can be *addressed* are inequities, through stating that “[…] our understanding was that we can address the inequality. These are addressable inequalities which are unfair and systematic and therefore they are inequities” (Informant 6). But again, this seemed to differ from another informant’s understanding—the same informant who noted differences for those from the U.K.—as they stated:

*“It’s like, there are differences between groups that when these differences are avoidable and unfair and they are, well, I believe that this is what you mean between inequities and inequalities, but we generally talk about inequalities. But sometimes there’s a terminology issue that some people use inequities to refer to unfair also differences”* (Informant 4).

(iii) And similarly, one informant seemed to understand the difference as being addressing inequalities involves a situation being made *equal* across individuals, whereas addressing inequities entails *addressing individual needs*. This is illustrated through the following quote:

*“I don’t know whether specific to Urban HEART inequality, like uh, inequalities people, you know, getting different, I mean, yeah, getting different things for the same situation, but whereas inequity is not getting, let’s say similar advantages. But, I mean inequity should be, equity should be based on needs, on particular needs, not just on things being equal, everybody getting an equal component of something. Whereas, so it goes deeper than equality and very, versus for inequity”* (Informant 11).

(iv) Aside from the use of “health inequity” and “health inequality,” two informants also differentiated between “equity” and “health equity.” Informant 1 nicely expressed this through stating, “I think equity is more important goal than health equity. It has a lot of other aspects: economic, social, cultural, environmental,” and that “we included some other aspects to make sure that health equity is respected actually to include other components as well.” This understanding is further broken down by Informant 12:

Well, I mean there’s health equity. So, in terms of how people, the general health of people is not equally distributed. Then there’s equity and health in terms of how equity more broadly in society impacts on health and vice versa. And then that leads us to the social determinants of health where inequity in social determinants resulted in equity and health outcomes as well.

### Health inequities in what?

A previous study investigating how the WHO discussed health equity in its key texts—the only study of this nature—found that the WHO referred to equity across various different aspects, including mortality and morbidity, healthcare utilization, allocation of resources, and opportunities [7].

In speaking to key informants, a similarly wide range of aspects emerged when speaking about equity: “[…] same access to budgets to […] health facilities or in terms of education, in terms of health insurance, in terms of other things, actually, economic or social facilities” (Informant 1); and “[…] equivalent access to health services and that things should be fairly distributed, whether it will be income, whether it’s like recognition, for example […]” (Informant 3).

However, it seems that access to healthcare services is prioritized. This is evident through Informant 13’s remarks that “[…] equitable access to opportunities for health, for healthcare, the full range of healthcare that has to be insured. So yeah, equity is a core of WHO’s work […]” and:

[…] in WHO we commonly used some of the models that explain inequities and access to healthcare […] these are all series or conceptual frameworks to understand inequalities in healthcare access and they identify factors like availability, affordability, accessibility of healthcare services.

## Discussion

The results of this study demonstrate that key informants involved with Urban HEART felt that equity was a core value in health and understood the broader concept of health inequity as avoidable, systematic, unnecessary, and unfair. However, some government discussions of health inequity were politically sensitive, with “unnecessary” being raised as debated by one government, and other governments unwilling to accept they have an inequitable society. Therefore, shirking their responsibilities and “requirement” to act accordingly, whether it be in disrupting systems of power, reallocating resources, or others. Maintaining the status quo is preferred to advance their own interests. This is a notable contrast to more recent discussions on inequity, which center around structural racism, colonialism, and relationships with land, as expressed in the work of the Pan American Health Organization’s Equity Commission [18], for example. When considering recent decolonization efforts in global health that are largely focused on mitigating prevailing gross power imbalances, select informants’ experienced challenges around the use of “unnecessary” paint a picture that this term may be disadvantageous to these ongoing decolonization efforts.

Overall, many informants felt the concept of health equity was quite vague, despite their expertise regarding Urban HEART. This recognized vagueness may play a role in many informants using the terms “health inequality,” “health inequity,” and “health equity” in differing ways. This could also be due to different uses of terms across countries, and perhaps differing country actors’ levels of understanding and emphasis given to social justice. Similarly, informants referred to health equity in terms of varying aspects, including access to budgets and healthcare services, which was seemingly prioritized. These findings demonstrate that the lack of clarity around health equity is pervasive, which is particularly alarming when noting that these key informants have extensive experience in health equity through their involvement with Urban HEART. These different understandings of the concept of health equity—whether due to misunderstandings or interpretation around meaning that is context- or linguistic-specific—can then be expected to transpire into different types of policy action. For instance, in considering how differently associated terminology is used and the lack of clarity around what health equity is referring to—whether it be prioritizing healthcare access or Urban HEART’s general focus on a range of SDH—it can be anticipated that stakeholders would be discussing and understanding very different things to be problems and imagining different solutions. While different understandings of solutions can be overcome when initiating planning, different understandings of health inequities may lead to the prioritization of only certain things, as defining health equity in a certain way may exclude certain aspects through measurement and assessment.

## Limitations

While these findings demonstrate how health equity was understood by key informants and how it was approached in Urban HEART, it is important to remain mindful that these are individual key informants who are relaying their perceptions. However, these perceptions yield insights into how health equity was understood in Urban HEART. Thus, these understandings—and lack of understandings—around health equity highlight areas for improvement in future health equity work.

Further, key informants’ responses may be biased, whether intentional or not, which may result in aspects that are not relayed. With numerous interviews being conducted and data saturation being reached, it is unlikely that individual unintentional omissions were missed. However, it is possible that intentional omissions were not expressed and therefore not reflected in this study. We can speculate that any potential intentional omissions may have been political in nature, following on from one informant, who is employed by the WHO, who indicated they hesitated to participate due to not being sure what they should be saying “on record.” This hesitancy signals how employees of large authoritative organizations like the WHO must be cautious of language employed, given the understanding that such discourses matter. Overall, it is these institutional discourses around health equity and how health equity is approached that shape broader practices, as evidenced by the WHO’s discourses shaping Urban HEART, subsequent uptake around the world, and translation to policies and practices.

## Conclusion

A key contribution from this study is the finding that despite the acceptance of health equity as being central, those who have experience with Urban HEART—which is focused on health equity—found the concept of health equity to be vague and defined key terms in different ways, despite recognizing key characteristics in the definition of health equity as outlined by Whitehead [17]. Moving forward, with the lack of shared understanding of what health equity entails, it will be important to establish a shared understanding across key terms prior to beginning any global health work, whether explicitly focused on health equity or not. This is essential for ensuring stakeholders approach policy or program development, implementation, monitoring, and evaluation from a similar perspective. Because different understandings can mean different health inequities are measured, and thus acted on, shared understandings are important for more deliberate action that seeks to address health inequities in a way that stakeholders envision. In the context of recent efforts to decolonize global health, having such discussions around what health equity entails is particularly important.

## Electronic supplementary material

Below is the link to the electronic supplementary material.

Supplementary Material 1

## Data Availability

The dataset generated and analyzed during this study is not publicly available due to the sensitive information contained and to ensure anonymity of key informants in accordance with the ethics protocol. Data may be available from the corresponding author on reasonable request and following ethical approval.

## Footnotes

1 Please note, this informant and others referred to Canada due to the interviewer’s geographical location, rather than them necessarily speaking about Urban HEART as employed in Canada.

## List of abbreviations

**SDH**: Social determinants of health
**Urban HEART**: Urban Health Equity Assessment and Response Tool
**WHO**: World Health Organization
**WKC**: WHO Kobe Centre
