# Supplementary material for: Probing key informants’ views of health equity within the World Health Organization’s Urban HEART initiative
Source: BMC Public Health. 2022 Oct 31;22:1989. doi: 10.1186/s12889-022-14395-z (PMC9620663; doi:10.1186/s12889-022-14395-z)
Supplement: Supplementary file 1 — Supplementary Material 1 [file 12889_2022_14395_MOESM1_ESM.docx]

1. Could you please confirm your:
   1. dates of involvement in Urban HEART,
   2. city, and
   3. role for Urban HEART (i.e. WHO Technical Officer or civil servant at what level of government)?
2. In your own work, how do you think about the relationship of equity and health?
3. Where does “equity” fit into the Urban HEART agenda?
4. In seeking to address health equity in Urban HEART, what do you think is the goal? In other words, were any aspects or components more important?
5. Are you familiar with any theories of justice or inequality?
   1. If yes, can you please explain your understanding?
   2. If yes, do you believe the WHO’s Urban HEART work aligns with any in particular?
6. Do you think Urban HEART worked to reduce inequity in the city?
   1. If yes or no, please explain.
7. Is your city still using Urban HEART?
   1. If no, why?
   2. If yes, what do you think has contributed to the sustainability of the initiative?
8. Why did your city decide to employ Urban HEART? In other words, what led to this being accepted within the government?
9. Were there any pivotal or influential moments while using Urban HEART in your city?

Do you know of any individuals that were involved in Urban HEART that I can contact as a potential participant? If so, do you mind providing me with their contact information?
